# Performance Analysis of Cooperative and Non-Cooperative Relaying over VLC Channels

**DOI:** 10.3390/s20133660

**Published:** 2020-06-30

**Authors:** Waled Gheth, Khaled M. Rabie, Bamidele Adebisi, Muhammad Ijaz, Georgina Harris

**Affiliations:** 1 Faculty of Science and Engineering, Manchester Metropolitan University, Manchester M15 6BH, UK; K.Rabie@mmu.ac.uk (K.M.R.); b.adebisi@mmu.ac.uk (B.A.); m.ijaz@mmu.ac.uk (M.I.); 2 School of Science, Engineering and Environment, University of Salford, Salford M5 4WT, UK; g.d.harris14@salford.ac.uk

**Keywords:** relaying protocols, cooperative relaying systems, energy efficiency, outage probability, visible-light communications (VLC)

## Abstract

The line-of-sight (LoS) channel is one of the requirements for efficient data transmission in visible-light communications (VLC), but this cannot always be guaranteed in indoor applications for a variety of reasons, such as moving objects and the layout of rooms. The relay-assisted VLC system is one of the techniques that can be used to address this issue and ensures seamless connectivity. This paper investigates the performance of half-duplex (HD) conventional DF relay system and cooperative systems (i.e., selective DF (SDF) and incremental DF (IDF)) over VLC channels in terms of outage probability and energy consumption. Analytical expressions for both outage probability and the minimum energy-per-bit performance of the aforementioned relaying systems are derived. Furthermore, Monte Carlo simulations are provided throughout the paper to validate the derived expressions. The results show that exploiting SDF and IDF relaying schemes can achieve approximately 25% and 15% outage probability enhancement compared to single-hop and DF protocols, respectively. The results also demonstrate that the performance of the single-hop VLC system deteriorates when the end-to-end distances become larger. For example, when the vertical distance is 3.5m, the single-hop approach consumes 20%, 40% and 45% more energy in comparison to the DF, SDF, and IDF approaches, respectively.

## 1. Introduction

Visible-light communication (VLC) is a last-mile access technology which uses visible light with wavelengths between 380 and 700 nm. This technology uses light-producing devices, such as light-emitting diodes LEDs, for the dual purpose of lighting and data transmission that can dramatically reduce cost and complexity. Another advantage of the VLC system is that it does not interfere with technologies in the already overcrowded radio frequency (RF) spectrum. It has potential as a green communication technology and can work complementarily with RF technology for indoor applications, such as providing network access at offices, homes, shopping centers, etc. [1,2,3]. Despite these advantages, connectivity disruption during the movement of the end-user is one of the major challenges of VLC technology. This is due to the short cell sizes of VLC links that require a frequent handover between VLC cells. Furthermore, light interference caused by the overlap of neighboring LEDs in the VLC environment can negatively affect the transmission over the VLC network [4,5,6]. Transmission failure can happen due to shadowing in VLC links. However, for better reliability and greater LEDs link coverage, different light sources in indoor environments, such as ceilings, desks, and floor lights are deployed as relay nodes [6,7,8].

Different relaying protocols, generally categorized into cooperative and non-cooperative, are often used in communication systems to ensure high performance and reliability. These protocols include amplify-and-forward (AF), compress-and-forward (CF), decode-and-forward (DF), selective DF (SDF), and incremental DF (IDF) relaying protocols. While the AF relay amplifies the received signal and forwards it to the end-user, the received signal is either decoded and forwarded by DF relays or compressed and forwarded by the CF relay to the destination. However, the cooperative version of DF protocols is known to be superior to the AF and DF protocols in terms of system performance and energy consumption [9]. However, this research work only considers SDF and IDF relay systems due its low complexity and simplicity for practical implementation in VLC. The authors of [9] discussed how the performance of the VLC can be improved by using light sources as DF and AF relay nodes in indoor environment. It was reported that the DF-based VLC system slightly outperforms the AF-based one. The authors in [10] investigated the possibility of deploying a mobile-user as AF or DF relay to assist the communications over VLC networks. It was revealed that DF-based systems offer greater improvement in the coverage area and bit-error-rate (BER) than that offered by the AF-based one. A cooperative non-orthogonal multiple access (NOMA)-based and DF-assisted VLC system was proposed by the authors of [11]. They concluded that the proposed system can enhance the network reliability and improve the network coverage.

Deployment of full-duplex AF and DF relays with VLC system was also discussed in [12]. The results showed that such deployment can significantly decrease the BER of the entire system. Furthermore, the capacity of the cooperative power line (PLC)/VLC communication can be improved by deploying AF relay, as presented in [6]. The authors showed that using AF relaying can increase the capacity of the system, particularly when the relay gain and transmit power are relatively high. A cascaded free-space optical (FSO)-VLC communication system in which the end-user is connected to the FSO back-haul link through a VLC link and DF relay was discussed in [13]. It was shown that the proposed system is feasible and highly efficient. The implementation of other relay schemes including SDF and IDF relaying was investigated in recent studies; see, e.g., [14,15]. The outcomes of these studies indicated that implementing such relaying protocols can improve the performance and enhance their reliability. It was also concluded that increasing the number of relays in the system can improve its performance in terms of outage probability but this will be at the cost of reducing the energy efficiency of the system [15].

Energy efficiency was investigated in previous studies [16,17,18,19]. Different techniques were discussed in [16,17] to improve the energy consumption in relay-based PLC systems. It was found by former authors that placing the DF relay at the mid-point between the source and destination with optimal timeshare gives the best energy efficiency performance. However, a completely different technique was proposed in [17], wherein the relay node harvests the power of the unwanted impulsive noise, which then contributes to powering the system. Harvesting energy from the first link and then using it as relay transmit power for the second link was discussed in [18,20] in which a cooperative relay-based VLC/RF communication system was considered. Furthermore, the energy harvesting (EH) technique where the energy from the VLC link is harvested and utilized as an additional energy resource for the DF relay was proposed in [19]. Improving energy efficiency and achieving better data rate by using hybrid VLC/RF links was investigated in [21], wherein the achieved outcomes were promising. An optimum EH time-switching protocol was proposed by the authors of [22,23] wherein the relay harvested the power of the useful signal and then utilized it to send this signal to its destination node.

Despite the considerable amount of published work in this area, to the best of the authors’ knowledge, no work in the open literature has provided a comprehensive performance analysis of multi-hop VLC systems in terms of outage probability and energy-efficiency. In contrast to the previous work which was limited to the use of conventional relays in VLC systems and in addition to our previous paper [24] which was limited to direct link and one relay analysis, the contributions of this article are as follows:

- A comprehensive study and analysis of outage probability and energy per bit consumption performance of multi-hop VLC networks. The single-hop scenario is also considered and investigated as a benchmark to compare with the cooperative systems.
- Derivation of accurate analytical expressions for the overall outage probability and energy-per-bit consumption of the proposed system configurations, including the single-hope and multi-hope approaches.
- Measure and study the effects of different parameters on the performance of the system, such as the number of relays on the network, source power and vertical distance of the VLC environment. Computer simulations are used to validate the theoretical results of the derived expressions.

Our contributions highlight the superiority of the VLC system with cooperative relaying protocols (i.e., IDF and SDF) over the single-hop and the conventional DF approaches. It is also shown that the vertical distance of the VLC environment can negatively affect both outage probability and energy consumption of the different system configurations which are considered in this paper.

The remainder of this paper is organized as follows. A full description of the proposed system model is presented in Section 2. The outage probability and energy per bit consumption are analyzed in detail for the different system configurations in Section 3. The numerical results of the analytical expressions and the computer simulations are discussed in Section 4. Finally, the main conclusions of this paper are drawn in Section 5.

## 2. System Model

**Figure 1:**
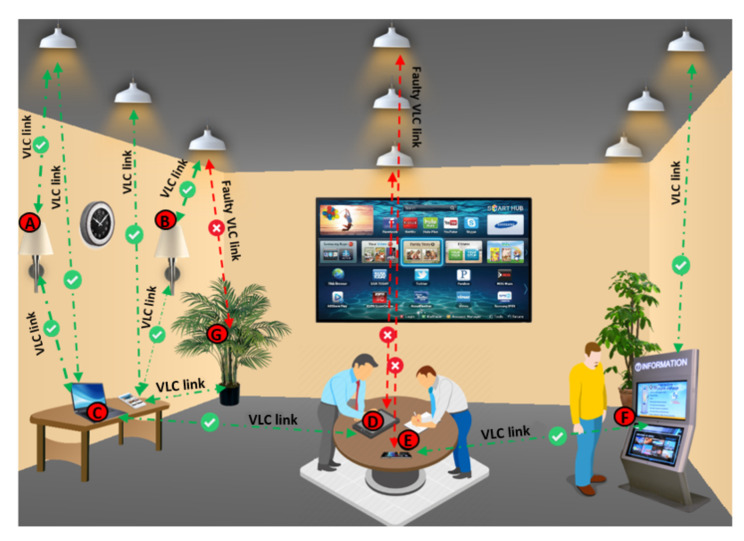
The proposed system model which consists of direct and relay nodes.

The system model of the proposed indoor multi-hop relaying VLC system is presented in Figure 1. The assumption is that LEDs which are the source data send the information directly to the destination through the VLC link. In case of transmission failure due to LED fault or shadowing issue, data is forwarded by relay nodes (i.e., intermediate light sources) to the destinations. In our case, nodes D and E lost communication due to faulty LEDs and shadowing, respectively. Therefore, these two destination nodes are connected to the source nodes through intermediate relay nodes (i.e., A, B, C and F relays).

**Figure 2:**
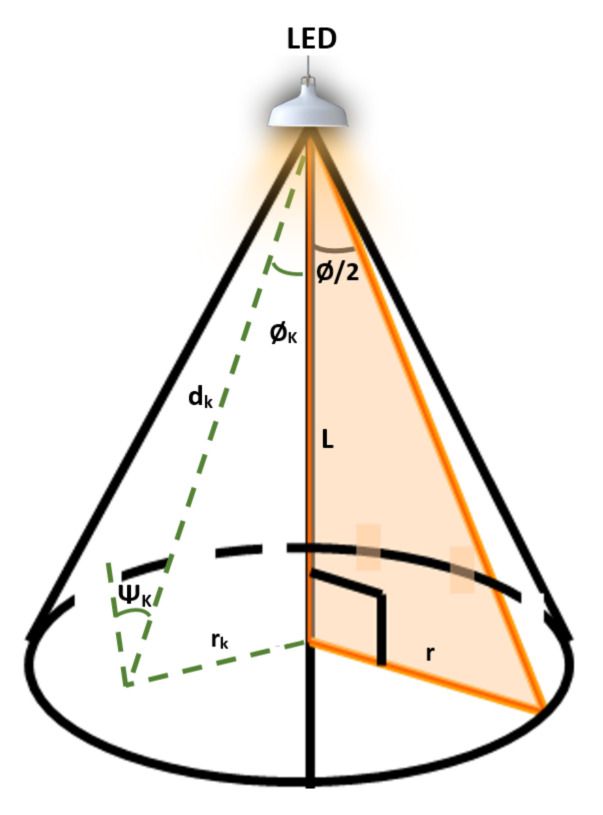
The line-of-sight channel of the VLC.

In this research work, only the line-of-sight (LoS) VLC channel is considered, as it represents more than 90% of the total received signal sent through the LED light [25]. The source nodes (the LEDs) are placed on the ceiling with Euclidean distances *d* to the destinations/relays and vertical distances *L* to the users/relays plane, as shown in Figure 2. It is assumed that the VLC links between the nodes are subjected to a random distribution which is affected by the uniform distribution of the location of the user [26,27,28]. For simpleness and without losing the generality, it is assumed that the noise over the VLC and Rf channels is additive white Gaussian noise (AWGN).

## 3. Performance Analysis

**Figure 3:**
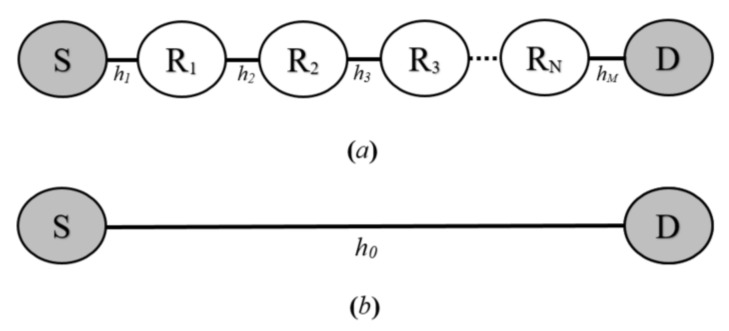
Basic block diagrams of the proposed VLC systems, (**a**) with N intermediate VLC relays and (**b**) with a direct VLC link.

The outage probability and energy efficiency performance of all of the proposed VLC system configurations are analyzed in this section. However, each configuration contains two nodes; namely, source (S) and destination (D) nodes. The communication between these two VLC nodes is achieved either via *N* intermediate relays, as shown in Figure 3a, or through a direct VLC link, as appears in Figure 3b. In the former configuration, the *n*th relay is denoted as $R_{n}$ where $n\in \left[1,N\right]$. On the other hand, in the single-phase configuration, end-to-end communication is accomplished without relaying.

### 3.1. Single-Hop VLC System

This system is a one-phase system where only two nodes are involved in the overall communication process; namely, source and destination modems. Hence, the energy-per-bit consumption for a single-hop VCL system can be expressed as:

(1) $$E_{b,SH}=\frac{P_{t,SH}}{R_{b}},$$

where $E_{b,SH}$ is the energy-per-bit consumption of the single-hop system, and $P_{t,SH}$ denotes the average optimal source power which is required to accomplish the desired outage probability for the single-phase approach. Here, $R_{b}$ represents the rate of the data which can be calculated by multiplying the bandwidth (B) and spectral efficiency (ε).

The overall outage probability of the direct link needs to be derived in order to determine $P_{t,SH}$. The outage probability of a communication system is the probability that the achieved instantaneous signal-to-noise ratio of the link is below the desired threshold. The received signal of a direct-link VLC link at the destination node $y_{d}$ is given as:

(2) $$y_{d}=\sqrt{P_{t,SH}}h_{0}s\left(t\right)+n,$$

where $h_{0}$ is the gain of direct channel, s(t) denotes the useful sent signal with E[s] = 1, and *n* represents the destination noise with variance $\sigma^{2}$ and zero mean.

The signal-to-noise ratio (SNR) at the destination node is given by:

(3) $$SNR=\frac{P_{t,SH}{\left|h_{0}\right|}^{2}}{\sigma^{2}}.$$

Using (3), the probability of the capacity of direct-link that is below the desired threshold of the information rate ω, can be expressed as:

(4) $$O_{SH}=\mathrm{Pr}\left\{\log_{2}\left(1+\mathrm{SNR}\right)<\omega \right\}.$$

This equation can be mathematically manipulated as:

(5) $$O_{SH}=\mathrm{Pr}\left\{SNR<(2^{\omega }-1)\right\}.$$

Here, (5) indicates the cumulative distribution function (CDF) of the VLC link which can also be written as:

(6) $$O_{SH}=F_{\gamma }\left(2^{\omega }-1\right),$$

where $F_{\gamma }\left(\cdot \right)$ is the CDF of the SNR.

Furthermore, in accordance with [6], the probability density function (PDF) of the instantaneous SNR of the VLC channel gain can be written as:

(7) $$f_{h_{k}^{2}}\left(t\right)=\frac{-Q^{\frac{2}{2+m_{k}}}{\left(\left(m_{k}+1\right)L^{m_{k}+1}\right)}^{\frac{2}{\left(m_{k}+3\right)}}t^{-\frac{m_{k}+5}{\left(m_{k}+3\right)}}}{\left(m_{k}+1\right)r^{2}},$$

(8) $$Q=\frac{1}{2\pi }AU\left(\phi_{K}\right)g\left(\phi_{K}\right)R_{ph},$$

where $t\in \left[C_{min},C_{max}\right]$, $C_{min}=\frac{{\left(Q\left(m_{k}+1\right)L^{m_{k}+1}\right)}^{2}}{{\left(r^{2}+L^{2}\right)}^{m_{k}+3}}$ and $C_{max}=\frac{{\left(Q\left(m_{k}+1\right)L^{m_{k}+1}\right)}^{2}}{L^{2(m_{k}+3)}}$, as indicated in [6,26]. *A* is the detector detection area; $U\left(\phi_{K}\right)$ and $g\left(\phi_{K}\right)$ are the optical filter and concentration gains, respectively; $R_{ph}$ indicates the responsivity of the photo-detector; *L* is the direct distance(s) from the LED to the user plane; *r* represents the maximum cell radius of the VLC environment; and $m_{k}$ is the order of the Lambertian radiation pattern, which is given by:

(9) $$m_{k}=\frac{-1}{\log_{2}\left(cos\left(\phi /2\right)\right)},$$

where ϕ/2 represents the semi-angle of the LED.

Hence, the CDF of direct VLC link can be calculated by integrating (7) over $\left[C_{min},C_{max}\right]$; hence the overall outage probability of the VLC link $O_{VLC}$ can be written as:

(10) $$O_{VLC}=\frac{-1}{r^{2}}{\left(\alpha QL^{\alpha }\right)}^{\frac{2}{\beta }}h^{-\frac{1}{\beta }}+\left(1+\frac{L^{2}}{r^{2}}\right),$$

where $\beta =m_{k}+3$ and $\alpha =m_{k}+1$.

Using (10), the end-to-end outage probabilityof the proposed single-hop approach can be calculated as:

(11) $$O_{SH}=\frac{-1}{r^{2}}{\left(\alpha QL_{SH}^{\alpha }\right)}^{\frac{2}{\beta }}{\left({\left|h_{0}\right|}^{2}\right)}^{-\frac{1}{\beta }}+\left(1+\frac{L_{SH}^{2}}{r^{2}}\right).$$

where $L_{SH}$ is the vertical distance of the direct link.

We now obtain $f_{{\left(h_{0}\right)}^{2}}F\left(\frac{\delta \sigma^{2}}{P_{t,SH}}\right)$.

(12) $$O_{SH}=\frac{-1}{r^{2}}{\left(\alpha QL_{SH}^{\alpha }\right)}^{\frac{2}{\beta }}{\left(\frac{\delta \sigma^{2}}{P_{t,SH}}\right)}^{\frac{-1}{\beta }}+\left(1+\frac{L_{SH}^{2}}{r^{2}}\right),$$

where $\delta =\left(2^{\omega }-1\right).$

By rearranging (12) and solving $P_{t,SH}$, we get

(13) $$P_{t,SH}={\left(\frac{{\left(\delta \sigma^{2}\right)}^{\frac{-1}{\beta }}{\left(\alpha QL_{SH}^{\alpha }\right)}^{\frac{2}{\beta }}}{-r^{2}O_{SH}+r^{2}+L_{SH}^{2}}\right)}^{-\beta }.$$

Finally, by substituting (13) into (1), the energy consumed per bit of the considered configuration can be obtained as:

(14) $$E_{SH}=\frac{1}{R_{b}}{\left(\frac{{\left(\delta \sigma^{2}\right)}^{\frac{-1}{\beta }}{\left(\alpha QL_{SH}^{\alpha }\right)}^{\frac{2}{\beta }}}{-r^{2}O_{SH}+r^{2}+L_{SH}^{2}}\right)}^{-\beta }.$$

### 3.2. Multi-Hop VLC System

In this subsection, both outage probability and energy efficiency of the different multi-hop relaying protocols are analyzed.

#### Decode-and-Forward Relaying Protocol

This is also called a non-cooperative DF configuration, where there is no direct link between the destination node and source node, and they only communicate through the DF relay which receives the data from the source then decodes and forwards it to the end-users. It is worth mentioning that the DF nodes are presumed to be positioned with equal distances between both ends the source and the destination nodes. However, it is more practical to have relays unevenly spaced between S and D nodes in many scenarios, and randomly spaced relay configurations are more practical. Mainly due to the complexity of analyzing such systems, we assumed equally spaced relays in this study. First, we derive the expressions for the cases when M = 2. This expression is a crucial part in our analysis because it allows us to determine the pattern of the generalized expression of the multi-hop scenario.

- Performance analysis for two links scenario M=2.

In such a configuration, the consumed energy is calculated as follows:

(15) $$E_{MH2}=\frac{P_{MH2}}{R_{b}}\left(O_{SR_{1}}+2O_{SR_{1}}^{c}\right),$$

where $P_{MH2}$ is the transmit power of the two-links system, $O_{SR_{1}}$ denotes the outage probability of the source-to-relay link and $O_{SR_{1}}^{c}$ is its complementary which is equal to $1-O_{SR_{1}}$.

For two link scenario, it is considered that the relay is placed at the half-distance between both end-nodes (i.e., $L_{SR_{1}}=L_{R_{1}D}$); the overall outage probability of this system can be expressed as:

(16) $$O_{2}=O_{SR_{1}}+O_{SR_{1}}^{c}O_{R_{1}D},$$

where $O_{R_{1}D}$ is the outage probability of the relay-to-destination link.

Now, assuming that source transmit power is equal to that of the DF relay (i.e., $P_{SR_{1}}=P_{R_{1}D}$), then following the same steps of subsection A, $O_{SR_{1}}$ and $O_{R_{1}D}$ can be defined as:

(17) $$O_{SR_{1}}=\frac{-1}{r^{2}}{\left(\alpha QL_{SR_{1}}^{\alpha }\right)}^{\frac{2}{\beta }}{\left(\frac{\delta \sigma_{r_{1}}^{2}}{P_{SR_{1}}}\right)}^{\frac{-1}{\beta }}+\left(1+\frac{L_{SR_{1}}^{2}}{r^{2}}\right),$$

(18) $$O_{R_{1}D}=\frac{-1}{r^{2}}{\left(\alpha QL_{R_{1}D}^{\alpha }\right)}^{\frac{2}{\beta }}{\left(\frac{\delta \sigma^{2}}{P_{R_{1}D}}\right)}^{\frac{-1}{\beta }}+\left(1+\frac{L_{R_{1}D}^{2}}{r^{2}}\right),$$

where $\sigma_{r_{1}}^{2}$ represents the variance of additive white Gaussian noise at the DF relay node. Both links of the considered DF-based system are identical, which means that the outage probabilities of both links are the same (i.e., $O_{SR_{1}}=O_{R_{1}D}$); thus, the outage probability of the entire system can be given as:

(19) $$O_{2}=O^{*}\left(2-\left(O^{*}\right)\right),$$

where $O^{*}=O_{SR_{1}}=O_{R_{1}D}$.

Substituting (17) and (18) into (19), the outage probability of the link can expressed as:

(20) $$\begin{array}{cc} O_{2} & =\left(\frac{-1}{r^{2}}{\left(\alpha QL_{2}^{\alpha }\right)}^{\frac{2}{\beta }}{\left(\frac{\delta \sigma_{2}^{2}}{P_{MH2}}\right)}^{\frac{-1}{\beta }}+\left(1+\frac{L_{2}^{2}}{r^{2}}\right)\right)\\ \; & \left(2-\left(\frac{-1}{r^{2}}{\left(\alpha QL_{2}^{\alpha }\right)}^{\frac{2}{\beta }}{\left(\frac{\delta \sigma_{2}^{2}}{P_{MH2}}\right)}^{\frac{-1}{\beta }}+\left(1+\frac{L_{2}^{2}}{r^{2}}\right)\right)\right), \end{array}$$

where $P_{MH2}=P_{SR_{1}}=P_{R_{1}D}$, $L_{2}=L_{SR_{1}}=L_{R_{1}D}$ and $\sigma_{2}^{2}=\sigma_{r_{1}}^{2}=\sigma^{2}$.

Using several basic algebraic manipulations to rearrange (20) and solving $P_{MH2}$, we obtain the optimal transmit power for the two-hop scenario, which can be defined as:

(21) $$P_{MH2}=\delta \sigma_{2}^{2}{\left(\frac{\left(1-{\left(1-O_{2}\right)}^{0.5}\right)-\left(1+\frac{L_{2}^{2}}{r^{2}}\right)}{\frac{-1}{r^{2}}{\left(\alpha QL_{2}^{\alpha }\right)}^{\frac{2}{\beta }}}\right)}^{\beta }.$$

Finally, by substituting (21) into (15), the energy consumption of the two-hop configuration can be obtained as:

(22) $$\begin{array}{cc} E_{MH2} & =\frac{1}{R_{b}}\left(\delta \sigma_{2}^{2}{\left(\frac{\left(1-{\left(1-O_{2}\right)}^{0.5}\right)-\left(1+\frac{L_{2}^{2}}{r^{2}}\right)}{\frac{-1}{r^{2}}{\left(\alpha QL_{2}^{\alpha }\right)}^{\frac{2}{\beta }}}\right)}^{\beta }\right)\\ \; & \;\;\;\;\;\left(O_{SR_{1}}+2O_{SR_{1}}^{c}\right). \end{array}$$

- Performance analysis with M-hops.

The overall outage probability of VLC system with M number of hops can be calculated as follows:

(23) $$\begin{array}{cc} O_{MH} & =O_{SR_{1}}+\left[\sum_{n=1}^{N-1}\left(O_{R_{n}R_{n+1}}\times \prod_{j=1}^{n-1}O_{R_{j}R_{j+1}}^{c}\right)\right.\\ \; & \;\;\left.+O_{R_{N}D}\times \prod_{n=1}^{N-1}O_{R_{n}R_{n+1}}^{c}\right]\times O_{SR_{1}}^{c}, \end{array}$$

where

(24) $$O_{SR_{1}}=\frac{-1}{r^{2}}{\left(\alpha QL_{SR_{1}}^{\alpha }\right)}^{\frac{2}{\beta }}{\left(\frac{\delta \sigma_{r_{1}}^{2}}{P_{SR_{1}}}\right)}^{\frac{-1}{\beta }}+\left(1+\frac{L_{SR_{1}}^{2}}{r^{2}}\right),$$

(25) $$\begin{array}{cc} O_{R_{n}R_{n+1}} & =\frac{-1}{r^{2}}{\left(\alpha QL_{R_{n}R_{n+1}}^{\alpha }\right)}^{\frac{2}{\beta }}{\left(\frac{\delta \sigma_{r_{1}}^{2}}{P_{R_{n}R_{n+1}}}\right)}^{\frac{-1}{\beta }}\\ \; & \;\;\;\;+\left(1+\frac{L_{R_{n}R_{n+1}}^{2}}{r^{2}}\right), \end{array}$$

(26) $$O_{R_{N}D}=\frac{-1}{r^{2}}{\left(\alpha QL_{R_{N}D}^{\alpha }\right)}^{\frac{2}{\beta }}{\left(\frac{\delta \sigma^{2}}{P_{R_{N}D}}\right)}^{\frac{-1}{\beta }}+\left(1+\frac{L_{RD}^{2}}{r^{2}}\right),$$

where *N* represents the number of relays on the network and n∈{1, 2, …, N}. Now, the optimal transmission power for a known outage probability can be given by:

(27) $$P_{MH}=\delta \sigma_{M}^{2}{\left(\frac{\left(1-{\left(1-O_{M}\right)}^{\frac{1}{M}}\right)-\left(1+\frac{L_{M}^{2}}{r^{2}}\right)}{\frac{-1}{r^{2}}{\left(\alpha QL_{M}^{\alpha }\right)}^{\frac{2}{\beta }}}\right)}^{\beta }.$$

The energy per bit consumption of the M-hope VLC system can be expressed as:

(28) $$\begin{array}{cc} E_{MH} & =\frac{P_{MH}}{R_{b}}(O_{SR_{1}}+O_{SR_{1}}^{c}\left[\sum_{n=1}^{N-1}(\left(n+1\right)O_{R_{n}R_{n+1}}\right.\\ \; & \prod_{j=1}^{n-1}O_{R_{j}R_{j+1}}^{c})\left.+O_{R_{N}D}\prod_{n=1}^{N-1}\left(N+1\right)O_{R_{n}R_{n+1}}^{c}\right]). \end{array}$$

### 3.3. Cooperative Relaying Protocols

The selective DF and the incremental DF are the two cooperative strategies of this relaying system. While the relay is always in a cooperative mode in the former configuration, it only cooperates in the latter one if the communication fails through the direct link.

#### 3.3.1. Selective DF Relaying Protocol

Two-time slots are involved in this relaying system. At the first time slot, the source sends the data to the cooperative relay and the destination nodes. At the second time slot, the DF relay decodes the received signal and forwards it to the destination node. However, in this protocol, both received signals at the destination (i.e, source signal and relay signal) are combined, which is called spatial diversity, which can considerably improve the performance of the communication systems that are based on this configuration [29]. In such scenarios, the consumed energy-per-bit is written as:

(29) $$E_{SDF}=O_{SR_{n}}\frac{P_{SDF}}{R_{b}}+\left(1-O_{SR_{n}}\right)\frac{2P_{SDF}}{R_{b}},$$

where $E_{SDF}$ denotes the energy-per-bit consumption of this SDF relaying and $P_{SDF}$ is the optimal transmit power. To began with, in order to defined the consumed energy in such configuration, we obtain the overall outage probability of this configuration which is expressed as:

(30) $$O_{SDF}=O_{SH}\left(O_{SR_{n}}+\left(1-O_{SR_{n}}\right)O_{R_{n}D}\right),$$

where $O_{SH}$ is the outage probability of the direct link given by (12); $O_{SR_{n}}$ and $O_{R_{n}D}$ are the outage probabilities of the first and second links, respectively, which can be written as:

(31) $$O_{SR_{n}}=\frac{-1}{r^{2}}{\left(\alpha QL_{SR_{n}}^{\alpha }\right)}^{\frac{2}{\beta }}{\left(\frac{\delta \sigma_{r_{n}}^{2}}{P_{SR_{n}}}\right)}^{\frac{-1}{\beta }}+\left(1+\frac{L_{SR_{n}}^{2}}{r^{2}}\right),$$

(32) $$O_{R_{n}D}=\frac{-1}{r^{2}}{\left(\alpha QL_{R_{n}D}^{\alpha }\right)}^{\frac{2}{\beta }}{\left(\frac{\delta \sigma^{2}}{P_{R_{n}D}}\right)}^{\frac{-1}{\beta }}+\left(1+\frac{L_{R_{n}D}^{2}}{r^{2}}\right),$$

where $L_{SR_{n}}$ is the length of the first link, $P_{SR_{n}}$ represents the minimum source power which is needed to accomplish $O_{SR_{n}}$, $L_{R_{n}D}$ indicates the second link length (i.e, relay-to-destination link) and is $P_{R_{n}D}$ the optimum SDF relay power which is required to achieve $O_{R_{n}D}$.

By keeping the assumption that the relay $R_{n}$ is placed at the mid-point between the source and the destination nodes, which provides the best performance of the SDF relay, the overall outage probability of the cooperative SDF relaying VLC system is simplified as:

(33) $$O_{SDF}=O_{SH}\left(O^{*}\left(2-O^{*}\right)\right),$$

where $O^{*}=O_{SR_{n}}=O_{R_{n}D}$.

Substituting (12), (31) and (32) into (33), the outage probability of the SDF relay is given in (34), as shown below:

(34) $$\begin{array}{cc} O_{SDF} & =\left(\frac{-1}{r^{2}}{\left(\alpha QL_{1}^{\alpha }\right)}^{\frac{2}{\beta }}{\left(\frac{\delta \sigma_{d}^{2}}{P_{SDF}}\right)}^{\frac{-1}{\beta }}+\left(1+\frac{L_{1}^{2}}{r^{2}}\right)\right)\left(\frac{-1}{r^{2}}{\left(\alpha QL_{2}^{\alpha }\right)}^{\frac{2}{\beta }}{\left(\frac{\delta \sigma_{d}^{2}}{P_{SDF}}\right)}^{\frac{-1}{\beta }}+\left(1+\frac{L_{2}^{2}}{r^{2}}\right)\right.\\ \; & \;\;\;\;\;\;\;\left.\left(2-\left(\frac{-1}{r^{2}}{\left(\alpha QL_{2}^{\alpha }\right)}^{\frac{2}{\beta }}{\left(\frac{\delta \sigma_{d}^{2}}{P_{SDF}}\right)}^{\frac{-1}{\beta }}+\left(1+\frac{L_{2}^{2}}{r^{2}}\right)\right)\right)\right) \end{array}$$

where $P_{SDF}=P_{SH}=P_{SR_{n}}=P_{R_{n}D}$, $L_{1}=L_{SH}=$ $2L_{2}=$ $2L_{SR_{n}}$ = $2L_{R_{n}D}$ and $\sigma_{d}^{2}=\sigma_{r}^{2}=\sigma^{2}$.

Now, numerical results for $P_{SDF}$ in (34), which is required to achieve the $O_{SDF}$, can be found by utilizing a software tool (specifically, a solve function in Mathematica software). Finally, substituting the numerical results of $P_{SDF}$ into (29), we obtain the consumed energy per bit performance of the proposed configuration.

#### 3.3.2. Incremental DF Relaying Protocol

As previously mentioned, compared to the SDF protocol where the relay is always in cooperative mode, the IDF only cooperates if the direct link between the source and destination does not meet the link quality requirement. This means that the relay does not take place in the communication process as long as the destination node receives the desired information from the source through the direct link. This can lead to decrease the consumed power and better energy efficiency [30]. In those scenarios, the consumed energy-per-bit is written as:

(35) $$\begin{array}{cc} E_{IDF} & =\left(1-O_{SD}\right)\frac{P_{IDF}}{R_{b}}+O_{SD}O_{SR_{n}}\frac{P_{IDF}}{R_{b}}\\ \; & \;\;+O_{SD}\left(1-O_{SR_{n}}\right)\frac{2P_{IDF}}{R_{b}}, \end{array}$$

where $E_{IDF}$ represents the energy consumption performance for the IDF configuration, $O_{SD}$ denotes the outage probability of the direct link which is equal to that of the single-hope one expressed in (12) and $P_{IDF}$ is the optimal transmit power which is required to fulfill the requirement of the outage probability of this approach. Each term of (35) terms refers to a distinct scenario. $\left(1-O_{SD}\right)\frac{P_{IDF}}{R_{b}}$—this term represents the consumed energy when the IDF relay does not cooperate in the communication process. The second one, $O_{SD}O_{SR_{n}}\frac{P_{IDF}}{R_{b}}$ depicts the energy consumption when he information signal can not be correctly decoded by both destination and IDF nodes. Here, the third term $O_{SD}\left(1-O_{SR_{n}}\right)\frac{2P_{IDF}}{R_{b}}$ refers to the consumed energy when the communication through the direct link fails and the IDF relay is in active mode.

Similarly to the outage probability of the SDF-based VLC system, the outage probability of the IDF one consists of three outage probabilities as:

(36) $$O_{IDF}=O_{SD}\left(O_{SR_{n}}+\left(1-O_{SR_{n}}\right)O_{R_{n}D}\right).$$

Substituting (12), (31) and (32) into (36), we can obtain the closed form of the outage probability of the IDF relaying VLC system which is equal to that of the SDF protocol represented in (34) at the top of this page. However, the numerical results of the $P_{IDF}$ can be straightforwardly determined by using the same software tools that were used to calculate the $P_{SDF}$ in the previous subsection. Finally, we substitute the values of $P_{IDF}$ into (35) to find the energy-per-bit consumption of the IDF relaying protocol.

## 4. Numerical Results and Discussions

The numerical results of the overall outage probabilities and the energy consumption for the different VLC system setups are presented and discussed in this section. Furthermore, Monte Carlo simulations are used in this section to validate these numerical results. The parameters of the proposed VLC system, unless specified otherwise, as shown in Table 1.

### 4.1. Average Outage Probability

**Figure 4:**
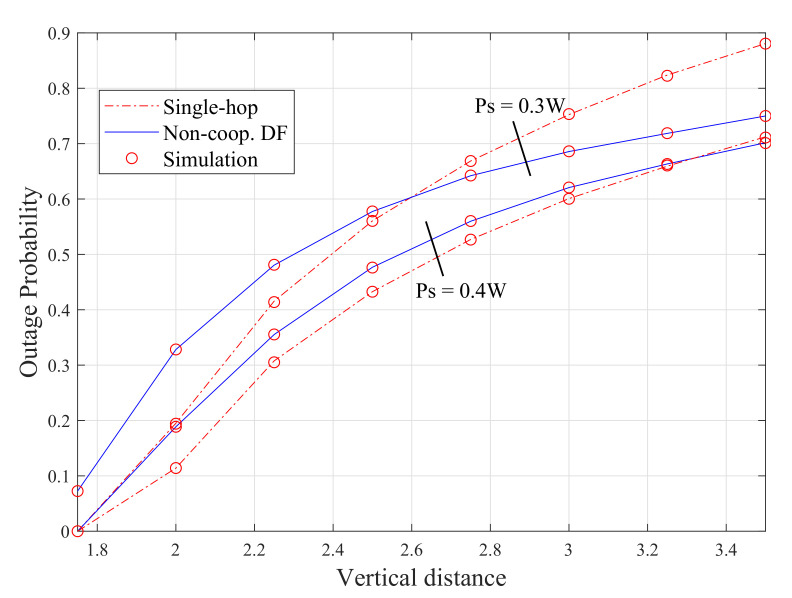
Outage probability of single-hop and non-cooperative DF relay configurations.

The performance of the different VLC system configurations is discussed in this subsection in terms of outage probability. The effect of different system parameters on its performance is also provided in this subsection. Figure 4 shows the outage probability for both the single-hop and the non-cooperative DF relay using (12) and (20), against the vertical distance for the source transmit power of 0.4 W and 0.3 W. It is noticeable, for both scenarios, that the numerical results of the outage probability for single-hop and two-hope links perfectly match with the simulation results. When the transmit power is 0.3 and the vertical distance is less than 2.6 m, it is clear that the single-hop approach outperforms the DF. This is because the DF relay operates in half-duplex (HD) mode, which leads to a substantial loss in spectral efficiency and thus increasing the outage probability of the system [31]. This implies that in short distances, when the direct link is available (i.e., the direct transmission is not affected by shadowing/blocking), using DF-assisted VLC systems becomes inefficient in terms of spectral efficiency. On the other hand, the outage probability of the DF configuration is 0.15% less than the single-hop approach when the vertical distance is 3.6 m for the same transmit power 0.3 W. This is because of the inverse proportional relationship between the system capacity and the source-to-destination distance in the direct link system.

It is also noticeable from this figure that the transmit power has a positive impact on the performance of both systems and the vertical distance can negatively affect the performance of both configurations. For example, in the single-hop scenario, the outage probability increases from 0 to 0.7 as the vertical distance changes from 1.6 to 3.6 m when the transmit power is 0.4 W, which represents a 70% increase. Furthermore, the outage probability is almost 0.9 when the vertical distance is 3.6 m and the transmission power is 0.3 W, whereas it is only 0.7 at the same vertical distance and the transmit power is 0.4 W.

**Figure 5:**
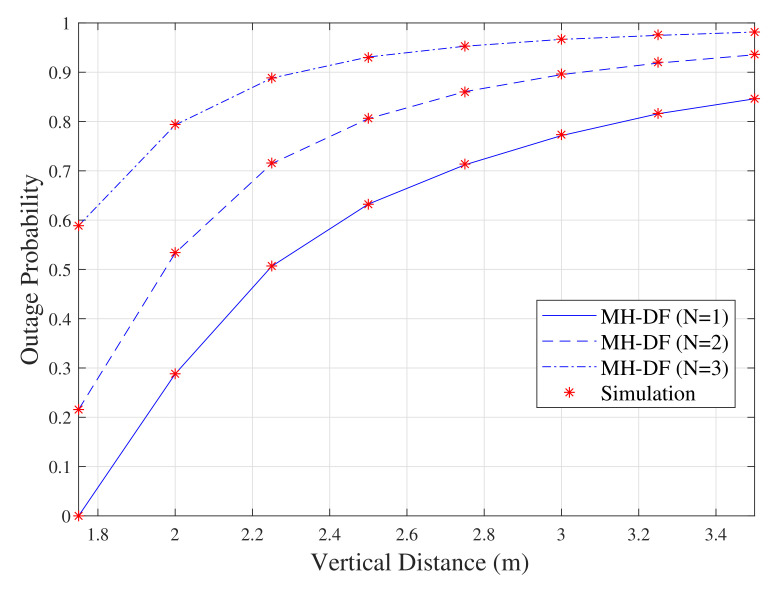
Outage probability of DF multi-hop scenarios (for N = 1, 2 and 3).

The analytical results of (20) and (23) are illustrated in Figure 5 along with the simulated results. The results show that increasing the vertical distance between the LED and the user plan always results in performance degradation for all of the system configurations. The results also show that the performance of this system setup (i.e., DF-based VLC system) is positively affected by the number of DF relays on the VLC system. For example, when the vertical distance is 3 m, the outage probabilities when N = 1, N = 2 and N = 3 are 0.77, 0.9 and 0.98, respectively.

**Figure 6:**
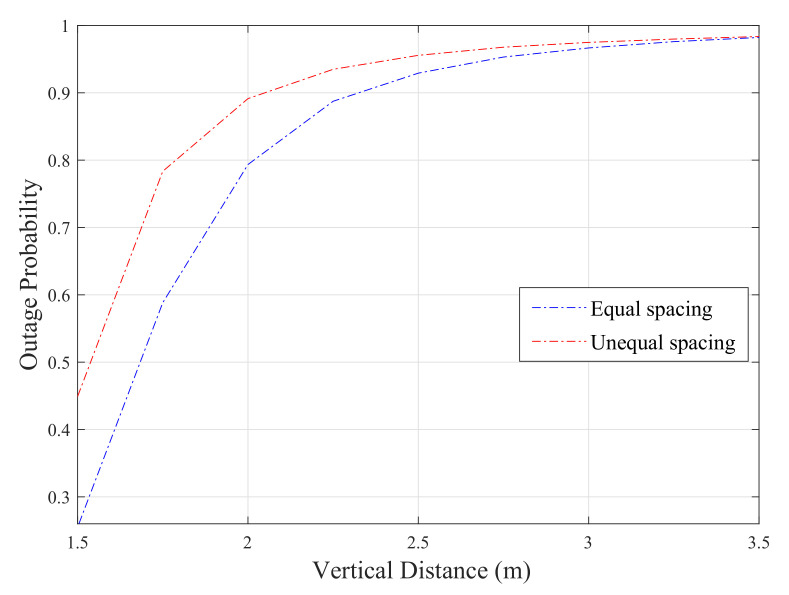
Simulated results of DF relay with N = 3.

Figure 6, represents simulated results for a MH-DF system with three relays. In the first scenario, the relays are evenly placed between the source and destination nodes (i.e., $L_{SR_{1}}=L_{R_{1}R_{2}}=L_{R_{2}R_{3}}=L_{R_{3}D}=$ 1 m). However, the relays are located with different distances from each other between both ends in the second scenario (i.e., $L_{SR_{1}}=$ 1 m, $L_{R_{1}R_{2}}=$ 1.5 m, $L_{R_{2}R_{3}}=$ 2 m, $L_{R_{3}D}=$ 0.5 m). The results show that the outage probability performance of the system is better when the relays are equally spaced between the source and destination than the unequal spacing for the same transmit power.

**Figure 7:**
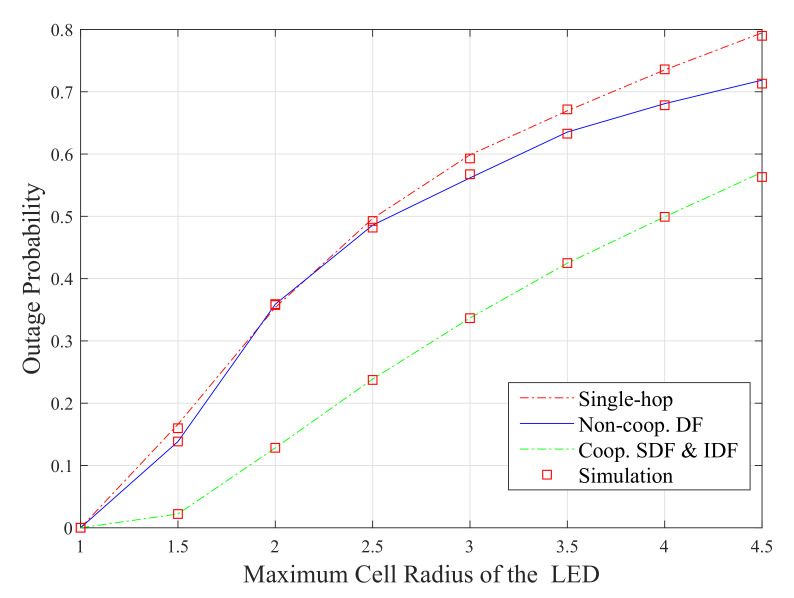
Performance comparison between the different VLC system setups as a function of VLC cell radius.

For the sake of performance comparison, the outage probabilities of the different configurations (i.e, the numerical results of (12), (20) and (34)) are compared and presented in Figure 7 as functions of the maximum cell radius of the VLC system. The results show that the performance of all of the considered VLC configurations degrades as the size of the cell radius of the LoS increases from 1 to 4.5 m. It can be seen from the figure that the cooperative DF setups (i.e., SDF and IDF) outperform the other two configurations (i.e., single-hop and DF-based ones). This is because, in cooperative protocols, the capacity of the communication system is substantially improved by the spatial diversity accomplished at the destination node by combining the signals received from the source node and the relay node [32]. When the maximum cell radius is 2 m, the outage probability of the cooperative DF relay scheme is 0.12 and it is almost 0.38 for both single-hop and DF approaches. However, the DF setup has the superior performance over the single-hop one for the higher values of the maximum cell radius of the VLC system (i.e, the maximum cell radius is higher than 2.5 m).

**Figure 8:**
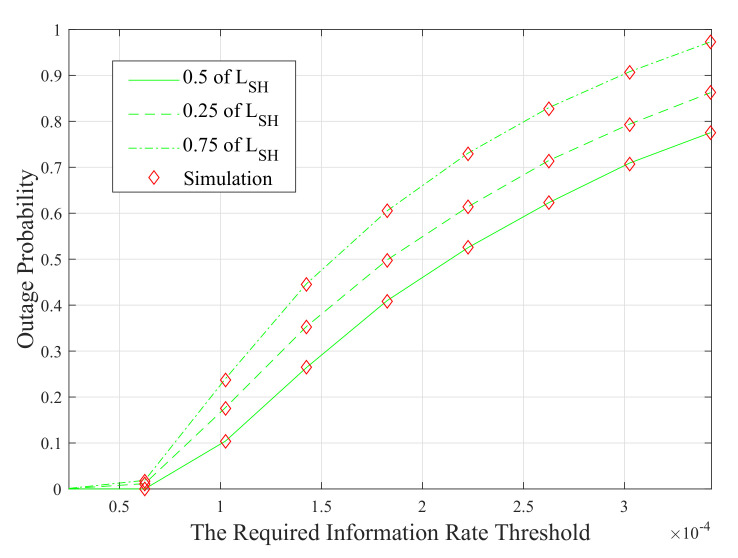
Average outage probability performance of the cooperative configurations as a function of the required information rate threshold values.

To illustrate the impact of the position of the cooperative DF relay on the performance of the system, the outage probability of this configuration is plotted versus the required information rate threshold in Figure 8.

It is clear from this figure that the system with the relay placed at the mid-point between the source and the destination nodes (i.e, $L_{RD}=L_{SR}=\frac{L_{SH}}{2}=2$ m) offers better performance than the other system setups. This is because relays perform better in symmetric systems. However, placing the cooperative relay closer to the source modem (i.e, $L_{SR}=0.25L_{SH}=1$ m) provides better performance than placing it after the mid-point between both nodes (i.e, $L_{SR}=3$ m).

### 4.2. Energy-Per-Bit Performance

**Figure 9:**
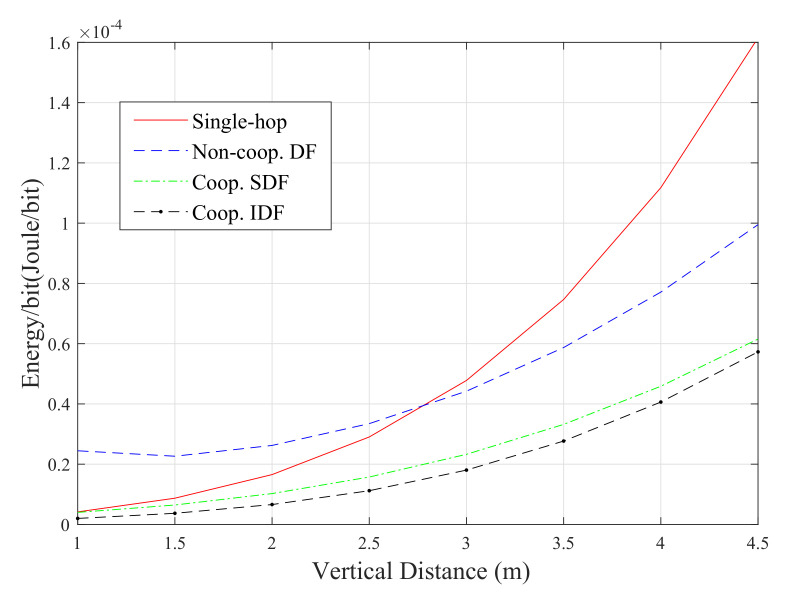
Energy performance comparison between the different VLC system setups.

The energy consumption of the proposed scenarios is discussed in this sub-section. First, for the sake of comparison, the energy consumption of the different system configurations which are considered in this paper (i.e, the analytical results of (14), (22), (29) and (35)) are plotted as a function of the vertical distance in Figure 9.

It is obvious from this figure that the IDF approach has superiority over the other relaying protocols in terms of energy consumption. For example, when the vertical distance is 4.5 m, it consumes almost 3%, 60% and 120% less energy compared to the SDF, DF and single-hop approaches, respectively. This can be simply explained by the fact that the DF relay in this system only cooperates when the communication through the direct link fails. However, the SDF scheme consumes less energy compared to both single-hop and DF-based systems. It is also noticeable that, for shorter distances (i.e., the vertical distance is less than 2.7 m), the single-hop approach is more energy-efficient than the DF one. The direct-link approach consumes about 10% and 1% less energy relative to the DF approach for vertical distances of 1 and 2.6 m, respectively. However, this configuration has almost the worst energy performance when the vertical distance is greater than 2.7 m. The other observation is that the consumed energy for all of the considered scenarios boosts when vertical distance becomes higher. This is because the energy consumption of the communication systems is inversely proportional to end-to-end distance.

**Figure 10:**
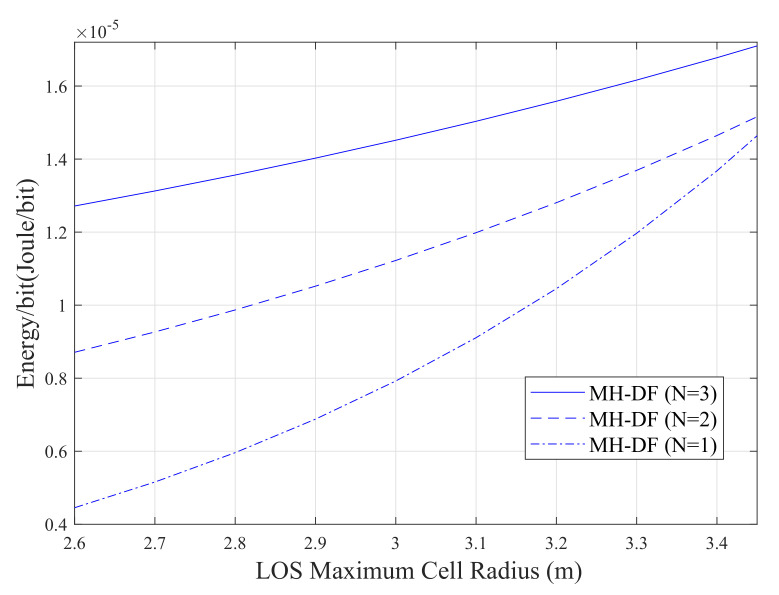
Energy-per-bit performance of the multi-hop system.

Figure 10 illustrates the effect of increasing the number of relays on the energy performance of the VLC system. The results show that as the number of relays increases, the system becomes more energy inefficient. This because of adding relays on the network contributes more to the total energy consumption of the system. However, it is evident that the system with three DF relays is the less energy-efficient one compared to the systems with two and one DF relays. For example, when the maximum cell radius is 3 m, this system consumes almost 20% and 45% more energy compared to that consumed by the system with two and one DF relays, respectively. It also can be seen that the systems consume more energy when the maximum cell radius of the LoS increases from 2.6 to 3.4 m.

**Figure 11:**
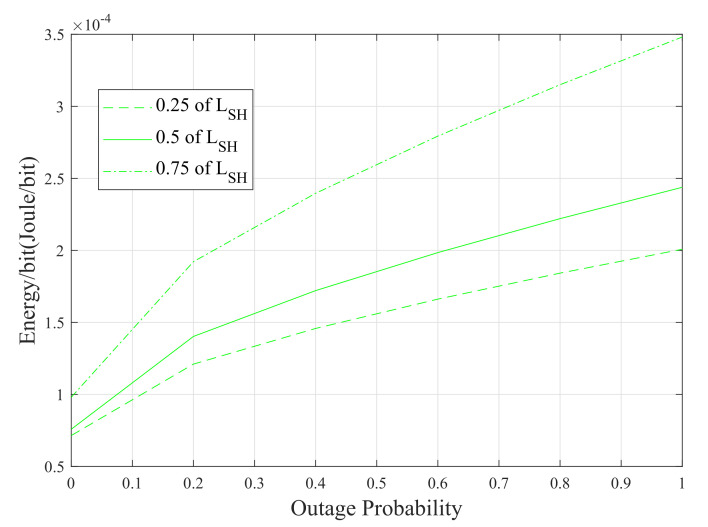
Energy consumption of the SDF system with respect to outage probability.

The last set of results of this paper is provided in Figure 11. The energy-per-bit consumption is plotted with respect to the outage probability of the SDF system for different source-to-relay distances. Although the SDF system with the relay placed at mid-point between the source and the destination modems (i.e, $L_{SR}=L_{RD}=2$ m) provides better performance in terms of outage probability, the system with the relay placed closer to the source (i.e, $L_{SR}=1$ m) consumes less energy. However, the energy consumed by the latter configuration is almost 30% less compared to the former one when the outage probability is 0.5. On the other hand, the system with $L_{SR}=2$ m outperforms the system with $L_{SR}=3$ m in terms of energy consumption.

## 5. Conclusions

This paper investigated and analyzed the performance of the relay-based VLC systems in terms of outage probability and energy consumption. Different relay protocols were considered; namely, multi-hop DF, SDF and IDF in addition to the single-hop approach. Accurate and close-forms for outage probability and the energy consumption of the different system setups were formulated and verified by Monte Carlo simulations. The derived expressions allow designers and engineers to optimize VLC network parameters such as the number of relays in the network, the distances between these relays and the optimum relay protocol for that specific practical system design. It was shown that the SDF and IDF protocols have superiority over the single-hop and multi-hop DF approaches in terms of outage probability and energy efficiency. However, the IDF configuration has the best energy consumption performance compared to the other VLC system configurations which were considered in this work. This is due to the fact that the IDF relay only takes part in the communication between the source and the destination nodes if the direct-link does not meet the required link quality. Our analyses also revealed that increasing the relay number on the network can dramatically improve the outage probability of the system but it contributes more to the energy consumption; thus, the system is less energy efficient. It is worth pointing out that other more sophisticated possibilities for cooperation, such as compress-and-forward and block Markov coding could offer higher transmission rates. However, such more sophisticated relaying approaches will likely be investigated in the future. For future work, the study will focus on implementing relays with VLC networks for outdoor applications such as road-to-vehicle, vehicle-to-vehicle and building-to-building communications. The analysis will take into consideration the effects of outdoor environmental factors such as sunlight, rain, fog and atmospheric disturbances.

## Figures and Tables

**Table 1:** System parameters.

Parameters	Values
$L_{SH}$	4 m
$L_{SR_{1}}=L_{R_{1}R_{2}}=L_{R_{n}R_{n+1}}=L_{R_{N}D}=$	$\frac{L_{SH}}{M}$
$P_{s}$	0.33 W
$A_{d}$	A=0.0001 m${}^{2}$
$U\left(\Psi_{K}\right)=g\left(\Psi_{K}\right)$	10 dB
$R_{p}$	1 A/W
$r_{e}$	3.6 m
ϕ/2	$60^{\circ }$
